# Using baseline MRI radiomics to predict the tumor shrinkage patterns in HR-Positive, HER2-Negative Breast Cancer

**DOI:** 10.3389/fonc.2025.1539644

**Published:** 2025-07-30

**Authors:** Lijia Wang, Yongchen Wang, Li Yang, Jialiang Ren, Qian Xu, Yingmin Zhai, Tao Zhou

**Affiliations:** ^1^ Department of Medical Imaging, The Fourth Hospital of Hebei Medical University, Shijiazhuang, Hebei, China; ^2^ Department of Breast Cancer Center, The Fourth Hospital of Hebei Medical University, Shijiazhuang, China; ^3^ Department of Pharmaceuticals Diagnostics, GE HealthCare, Beijing, China

**Keywords:** breast cancer, neoadjuvant chemotherapy, tumor shrinkage patterns, radiomics, MRI

## Abstract

**Introduction:**

This study aimed to develop and validate a predictive model for tumor shrinkage patterns in hormone receptor-positive, HER2-negative (HR+/HER2-) breast cancer patients undergoing neoadjuvant chemotherapy (NAC).

**Methods:**

A retrospective analysis was conducted on 227 HR+/HER2- breast cancer patients with a desire for breast conservation, examining their clinicopathological characteristics, traditional MRI features, and radiomics features. Patients were divided into training and validation cohorts in a 7:3 ratio. Tumor shrinkage patterns were classified into Type I and Type II based on RECIST 1.1 criteria. A clinical model was established using Ki67 quantification and enhancement pattern. Radiomics features were extracted and analyzed using machine learning algorithms, including Logistic Regression (LR), Support Vector Machine (SVM), Decision Tree (DT), and Random Forest (RF). A combined clinical-radiomics model was also developed.

**Results:**

The clinical model achieved an area under the curve (AUC) of 0.624 in the training cohort and 0.551 in the validation cohort. The RF radiomics model showed the highest predictive performance with an AUC of 0.826 in the training cohort and 0.808 in the validation cohort. The combined clinical-radiomics model further improved prediction accuracy, with an AUC of 0.831 in the training cohort and 0.810 in the validation cohort.

**Conclusion:**

Radiomics features based on baseline MRI significantly enhance the prediction of tumor shrinkage patterns in HR+/HER2- breast cancer patients. This approach aids in the early identification of patients likely to benefit from breast-conserving surgery and facilitates timely treatment adjustments.

## Introduction

Breast Cancer has ranked first in the incidence of malignant tumors among women worldwide for ten consecutive years. In 2023, there were over 2.3 million new cases and more than 670,000 deaths worldwide seriously threatening the health and lives of women (1). Breast at present, the research hotspots of breast cancer still lie in the differentiation of benign and malignant BC (2) and the evaluation of treatment efficacy, etc. Neoadjuvant chemotherapy (NAC) has emerged as a crucial treatment modality for locally advanced breast cancer (LABC), especially in triple negative and human epidermal growth factor receptor 2 (HER2) positive BC, by reducing tumor burden, decreasing tumor stage, and increasing the possibility of breast-conserving surgery (3, 4). Typically, patients who achieve a complete response (CR) or exhibit a simple centripetal shrinkage pattern after NAC are more likely to meet the negative margin requirements for breast-conserving surgery. Conversely, centripetal shrinkage accompanied by satellite lesions or fragmentation may result in positive margins or an increased recurrence rate (5). Hormone receptor (HR)-positive and human epidermal growth factor receptor 2 (HER2)-negative (HR+/HER2-) breast cancer accounts for 65-70% of BC cases (6). Compared to other molecular subtypes, patients with HR+/HER2- BC have lower rates of achieving pathologic complete response (pCR) and objective response after NAC, making it challenging for some patients to benefit from NAC (7). Therefore, early and accurate prediction of tumor shrinkage patterns in HR+/HER2- BC patients after NAC is critical for ensuring patients benefit from NAC with the goal of breast conservation while avoiding overtreatment and ineffective therapy.

Magnetic resonance imaging (MRI) is currently widely acknowledged and utilized by clinicians due to its exceptional soft tissue resolution and multifunctional imaging sequences. A single scan can provide the following critical information: the exact size, three-dimensional morphology, and spatial distribution characteristics of breast tumors; the anatomical relationship between the tumor and surrounding structures (e.g., pectoral muscles and skin); dynamic enhancement patterns; and the degree of restricted diffusion of water molecules, among others. Additionally, MRI facilitates a comprehensive evaluation of regional lymph node status (8). Radiomics, by deeply mining a large number of imperceptible features within traditional imaging, can screen for high-dimensional features with high stability and reproducibility to establish predictive models based on different clinical problems. Existing studies have applied radiomics to BC molecular subtyping (9), lymph node status (10), and NAC efficacy evaluation (11). At present, some studies attempt to combine MRI with radiomics to predict the contraction pattern of tumors after neoadjuvant therapy for breast cancer. However, these studies either classified the contraction patterns of tumors into complex types 4-5, or failed to distinguish the subtypes of breast cancer. However, studies specifically targeting the simple tumor shrinkage patterns after NAC in HR+/HER2 BC patients who are insensitive to chemotherapy are limited (12).

This study aims to retrospectively analyze the clinicopathological characteristics, traditional MRI features, and radiomics features of HR+/HER2- cancer BC patients to identify features independently associated with tumor shrinkage patterns post-NAC and establish a predictive model. The goal is to identify patients who can undergo breast-conserving surgery post-shrinkage and those with poor shrinkage patterns early, to adjust treatment strategies in a timely manner.

## Materials and methods

### Study participants

This study was approved by the Ethic Committee of the Fourth Hospital of Hebei Medical University (2022037). The requirement for informed consent was waived by the Ethics Committee of the Fourth Hospital of Hebei Medical University because of the retrospective nature of the study.

We retrospectively analyzed the medical records of breast cancer patients who received NAC treatment and surgical resection at our center. Inclusion criteria were: (1) female patients with a desire for breast conservation, diagnosed with invasive breast cancer by core needle biopsy, and confirmed as HR+ (ER or PR positive)/HER2- by immunohistochemistry (IHC) or fluorescence *in situ* hybridization (FISH) with no distant metastasis; (2) complete clinical data; (3) All MRI examinations were scheduled before needle biopsy, complete breast MRI examinations before and after NAC; (4) received at least six cycles of NAC before surgery, and complete pathological data post-surgery. Exclusion criteria included patients with bilateral breast cancer and those with breast cancer during pregnancy. Patients were randomly divided into a training cohort and a validation cohort in a 7:3 ratio (13, 14).

### MRI image acquisition

All patients underwent dynamic contrast-enhanced MRI (DCE-MRI) of the breast before and after NAC. The examinations were performed using a GE Hde 1.5T superconducting MRI scanner with an 8-channel breast coil, with patients in the prone position. The MRI protocol included axial T1-weighted images without fat suppression (TR/TE 360/7 ms, field of view [FOV] 32 cm, matrix 192×192 mm, thickness 5 mm), T2-weighted images with fat suppression (TR/TE 8240/80 ms, FOV 32 cm, matrix 320×240 mm, thickness 5 mm), diffusion-weighted imaging (DWI) (b=0, 800; TR/TE 6500/85 ms, thickness 5 mm, matrix 128×128 mm), and apparent diffusion coefficient (ADC) mapping. Gadolinium contrast agent (Gadodiamide, Omniscan^®^, GE Healthcare) was administered via a high-pressure injector into the antecubital vein at a dose of 0.1 mmol/kg and a rate of 2.5 ml/s, followed by a 10 ml saline flush at the same rate. Dynamic contrast-enhanced scans were performed starting 18 seconds after injection of the contrast agent (VIBRANT: TR/TE 5.6/2.4, matrix 320×288 mm, thickness 2 mm, 61 s/phase, 1 pre-contrast and 7 post-contrast phases).

### Determining shrinkage patterns

**Figure 1:**
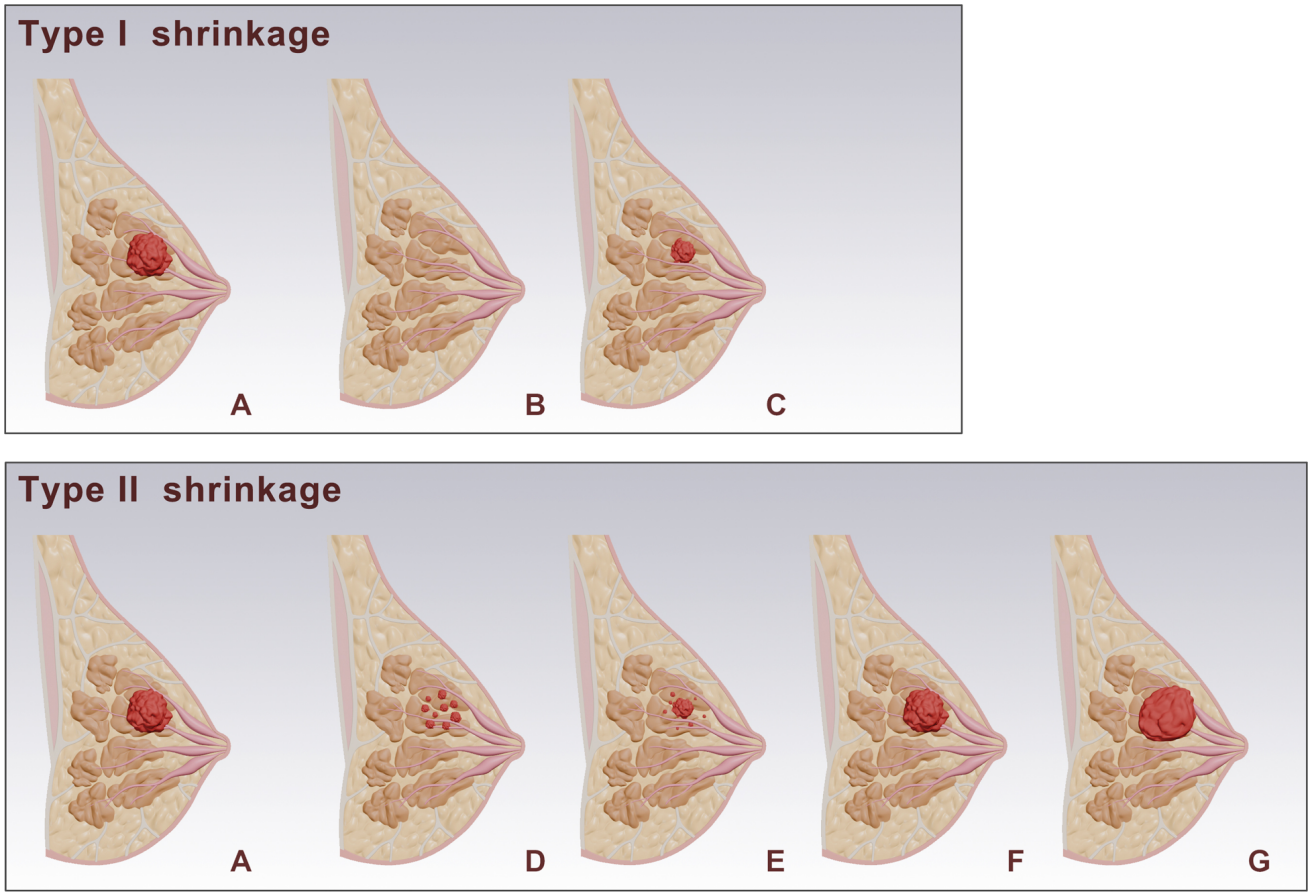
Schematic representation of breast tumor responses after NAC. **(A)** shows the baseline tumor. Post-NAC responses are categorized into Type I and Type II shrinkage. Type I shrinkage includes: **(B)** complete response: where no residual tumor is visible. **(C)** Simple concentric shrinkage: where the tumor mass decreases in size while maintaining its original shape. Type II shrinkage includes: **(D)** Tumor fragmentation: The tumor splits into multiple discontinuous lesions, while maintaining approximately the same overall extent **(E)** Concentric shrinkage with satellite lesions: The tumor mass contracts inward as a whole, with residual lesions remaining in the periphery. **(F)** Stable: where there is no significant change in tumor size. **(G)** progression: where there is an increase in tumor size.

Two radiologists (Readers 1 and 2, who have 5 and 13 years of experience in breast MRI diagnosis, respectively) re-read the routine breast MRI images, image features and clinical information were recorded. Based on MRI imaging before and after NAC, and according to the Response Evaluation Criteria in Solid Tumors version 1.1 (RECIST 1.1), tumor shrinkage patterns were classified into Type I and Type II shrinkage. Type I shrinkage included complete response (CR) and concentric shrinkage. CR was defined as no residual tumor visible on imaging. Concentric shrinkage was defined as a residual solitary tumor with a longest diameter reduction of ≥30% from baseline. All other shrinkage patterns were classified as Type II shrinkage (**Figure 1**). The determination of shrinkage patterns was independently performed by two radiologists with 5 and 15 years of experience in breast MRI diagnosis, respectively. In cases of disagreement, a consensus was reached through discussion.

### Development of the clinical model

The clinical and pathological characteristics of the patients were recorded, including age, menstrual status, tumor marker levels (CEA, CA125, CA153), tumor size, presence of skin invasion, presence of lymph node metastasis, estrogen receptor (ER) and progesterone receptor (PR) status, HR and HER2 status, and Ki67 quantification and grading. All pathological features were based on pre-NAC core needle biopsy results, evaluated by qualified and experienced oncology pathologist. Traditional imaging features included tumor morphology, tumor longest diameter, tumor margins, enhancement pattern, type of time-signal intensity curve (TIC), early enhancement rate, and ADC value. The formula for calculating the early enhancement rate was as follows:


$$Early\;enhacement\;rate=\frac{\left(SI_{post}-SI_{pre}\right)}{SI_{pre}}\times 100\%$$

Where $SI_{pre}$ and $SI_{post}$ were the tumor signal intensities before and at the second post-contrast phase (120 seconds after injection), respectively.

Univariable and multivariable analyses were performed on the clinical and pathological characteristics and traditional imaging features to identify independent predictive factors associated with tumor shrinkage patterns, and to establish the clinical model.

### Development of the radiomics model

Semi-automated 3D tumor segmentation was performed on the first post-contrast axial images using Slicer software (version 5.6.1, https://www.slicer.org). Initial segmentation was conducted by a junior radiologist and subsequently reviewed and refined by a senior radiologist to ensure accuracy. Prior to feature extraction, images underwent preprocessing, including linear interpolation resampling to 1×1×1 mm³ voxel size, z-score normalization of image intensities, and discretization to 5 gray levels. Radiomic features were extracted using the PyRadiomics package, encompassing morphological features, first-order statistical features, texture features, and higher-order filtered features. The feature selection process was as follows (1): each feature underwent univariate analysis using the Mann-Whitney U test, retaining features with *P*<0.05to ensure significant differences between tumor shrinkage patterns; (2) spearmancorrelation coefficients were calculated between features, and features with ρ >0.8 were removed to avoid multicollinearity; (3) The Boruta algorithm was employed to select the most predictive features through iterative analysis of feature importance scores (15) (4).a 5-fold cross-validation procedure exclusively within the training set (**Supplementary Figure S1**).

Using the selected radiomic features, models were constructed with the following machine learning algorithms: Logistic Regression (LR), Support Vector Machine (SVM), Decision Tree (DT), and Random Forest (RF). The model with the best predictive performance was chosen as the optimal radiomics model, producing radiomics scores (Rad-score). A combined clinical-radiomics model was developed by performing multivariate stepwise logistic regression analysis on the independent predictive factors from the clinical model and the Rad-score.

### Statistical analysis

Statistical analysis was conducted using SPSS software (version 21.0) and R software (version 4.2.0). Categorical variables were analyzed using the chi-square test or Fisher’s exact test, and continuous variables were analyzed using independent sample *t*-tests or Mann-Whitney *U* tests, depending on the normality of the data distribution. The performance of the models was evaluated and compared using receiver operating characteristic (ROC) curves and the DeLong test. Calibration performance was assessed using calibration curves, and clinical net benefit was evaluated using decision curve analysis (DCA). A two-tailed*P*<0.05 was considered statistically significant.

## Result

### Patient characteristics

A total of 227 patients were included in this study, with a mean age of 49.78 ± 10.93 years (range 23–72 years). After NAC, 122 patients (53.7%) exhibited Type I shrinkage, including 12 cases of imaging CR, while 105 patients (46.3%) exhibited Type II shrinkage. Patients were randomly divided into a training cohort (N=160) and a validation cohort (N=67) in a 7:3 ratio. There were no statistically significant differences in clinicopathological characteristics and traditional imaging features between the training and validation cohort.

### Clinical model

Univariable analysis of the training cohort data revealed that differences in the presence of skin invasion, Ki67 quantification, and enhancement pattern between the Type I and Type II shrinkage groups were statistically significant (all *p*<0.05) (**Table 1**). Multivariable stepwise regression analysis indicated that Ki67 quantification was an independent predictive factor for tumor shrinkage pattern (**Table 2**). Therefore, a clinical model predicting tumor shrinkage patterns after NAC in HR+/HER2- breast cancer was established based on Ki67 quantification and enhancement pattern. The area under the curve (AUC) for this clinical model was 0.624 (95% CI: 0.539-0.709) in the training cohort and 0.551 (95% CI: 0.412-0.689) in the validation cohort.

**Table 1:** Univariable analysis of clinicopathological characteristics between Type I and Type II shrinkage groups.

Characteristics	Type I shrinkage	Type II shrinkage	*P*
	N=86	N=74	
Age			
*M* [*Q*_1_;*Q*_3_]	50 [41;59]	51 [42;58]	0.858
Menstrual			
Postmenopausal	36 (41.860%)	34 (45.946%)	Ref.
Premenopausal	50 (58.140%)	40 (54.054%)	0.608
CEA			
Normal	81 (94.186%)	70 (94.595%)	Ref.
Abnormal	5 (5.814%)	4 (5.405%)	0.922
CA125			
Normal	72 (83.721%)	63 (85.135%)	Ref.
Abnormal	14 (16.279%)	11 (14.865%)	0.813
CA153			
Normal	73 (84.884%)	60 (81.081%)	Ref.
Abnormal	13 (15.116%)	14 (18.919%)	0.530
Skin invasion			
No	80 (93.023%)	61 (82.432%)	Ref.
Yes	6 (6.977%)	13 (17.568%)	0.044
Lymph node metastasis			
No	7 (8.140%)	6 (8.108%)	Ref.
Yes	79 (91.860%)	68 (91.892%)	0.999
ER			
*M* [*Q*_1_;*Q*_3_]	90% [80%;90%]	90% [80%;90%]	0.153
PR			
*M* [*Q*_1_;*Q*_3_]	60% [30%;80%]	55% [11%;80%]	0.806
HR			
Single positive	10 (11.628%)	6 (8.108%)	Ref.
Double positive	76 (88.372%)	68 (91.892%)	0.477
HER2			
None expression	21 (24.419%)	15 (20.270%)	Ref.
Low expression	65 (75.581%)	59 (79.730%)	0.540
Ki67quantification			
*M* [*Q*_1_;*Q*_3_]	30% [20%;40%]	20% [20%;40%]	0.018
Ki67grading			
<30%	8 (9.302%)	12 (16.216%)	Ref.
≥30%	24 (27.907%)	27 (36.486%)	0.052
Tumor size			
≤2cm	20 (23.256%)	10 (13.514%)	Ref.
>2cm&≤5cm	56 (65.116%)	57 (77.027%)	0.100
>5cm	10 (11.628%)	7 (9.459%)	0.606
Tumor shape			
oval	52 (60.465%)	35 (47.297%)	Ref.
Irregular	34 (39.535%)	39 (52.703%)	0.099
Tumor diameter (cm)			
*M* [*Q*_1_;*Q*_3_]	2.930 [2.330;3.815]	2.995 [2.305;3.723]	0.954
Tumor margin			
Clear	18 (20.930%)	12 (16.216%)	Ref.
Not circumscribed	68 (79.070%)	62 (83.784%)	0.457
Enhancement pattern			
Homogeneous	12 (13.953%)	3 (4.054%)	Ref.
heterogeneous	74 (86.047%)	71 (95.946%)	0.034
TIC type			
Inflow type	2 (2.326%)	2 (2.703%)	Ref.
Plateau type	27 (31.395%)	20 (27.027%)	0.791
Outflow type	57 (66.279%)	52 (70.270%)	0.933
Early enhancement rate			
*M* [*Q*_1_;*Q*_3_]	134.5% [110.2%;168.4%]	142.6% [111.1%;180.7%]	0.304
ADC			
*M* [*Q*_1_;*Q*_3_]	0.951 [0.810;1.067]	0.957 [0.863;1.090]	0.342

**Table 2:** Multivariable analysis results of the clinical model and the combined model.

Variable	Clinical model			Combined model		
	*β*	OR (95%CI)	*P*	*β*	OR (95%CI)	*P*
Intercept	-0.626		0.392	-3.017		<0.001
Ki67quantification	-2.113	0.121 (0.016-0.789)	0.032	-2.196	0.111 (0.009-1.176)	0.076
Enhancement pattern	1.216	3.375 (0.997-15.455)	0.072	0.479	1.615 (0.400-8.236)	0.523
Rad-score				7.041	1142.914 (127.805-14940.097)	<0.001

### Radiomics model

**Figure 2:**
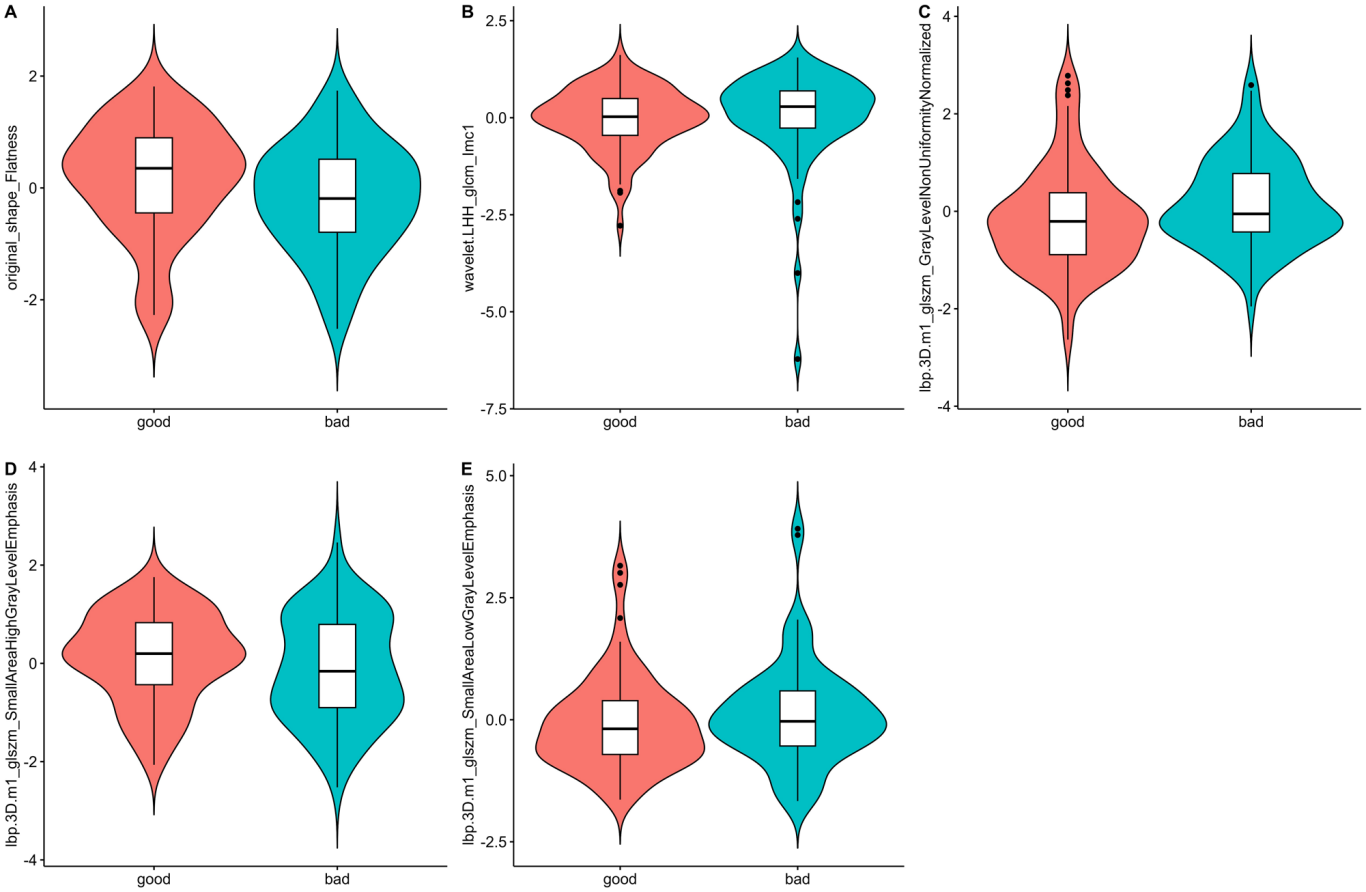
Violin plots of the radiomics features. The distribution of five key radiomics features in the good and bad shrinkage groups after NAC. **(A–E)** represent different radiomics features, highlighting their respective distributions and variations between the two groups of Type I and Type II shrinkage.

**Figure 3:**
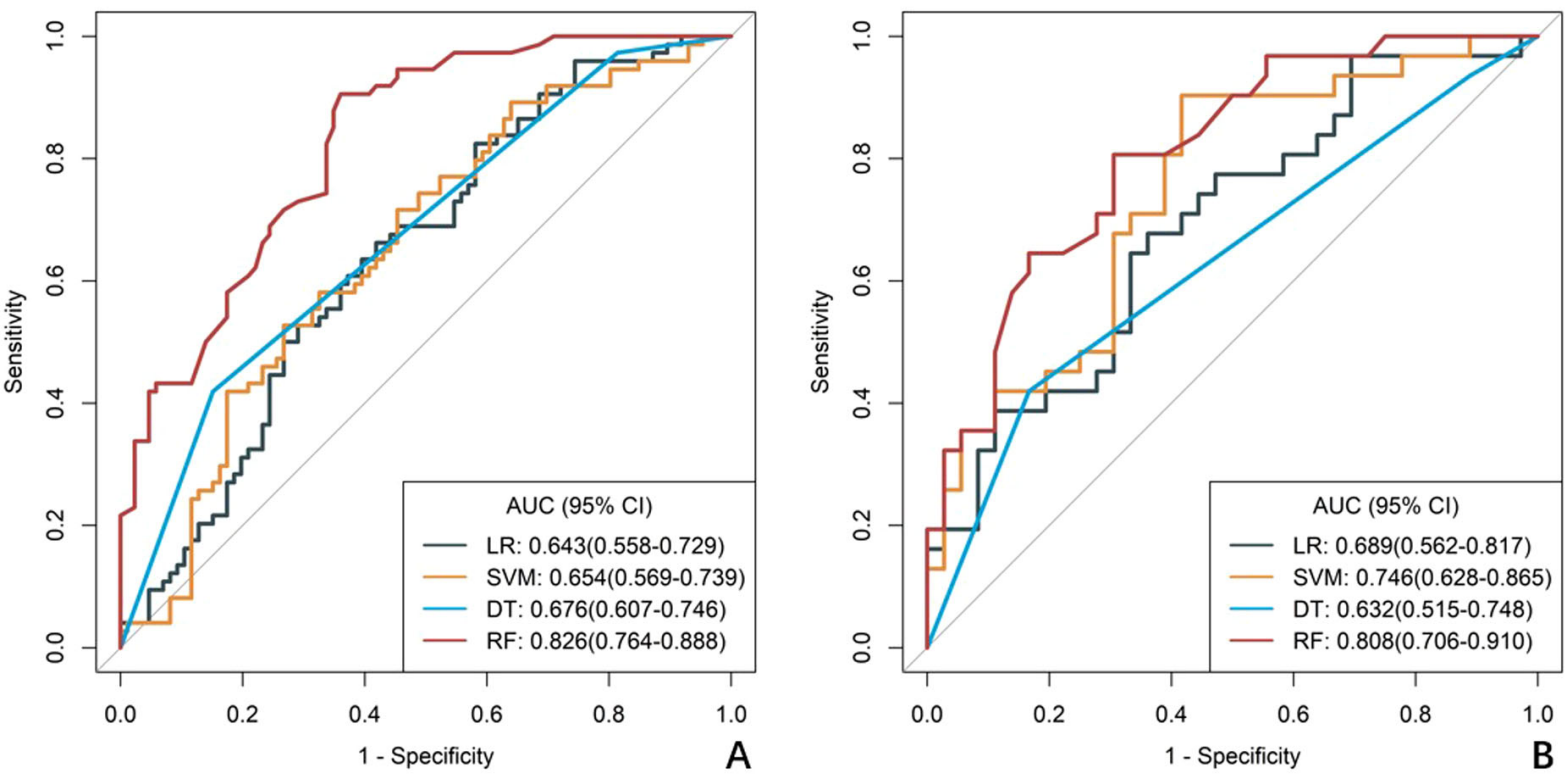
ROC curves of radiomics models constructed using four machine learning algorithms. **(A)** the training cohort, **(B)** the validation cohort. The curves illustrate the predictive performance of models developed with Logistic Regression (LR), Support Vector Machine (SVM), Decision Tree (DT), and Random Forest (RF) algorithms .RF curve shows best balance of sensitivity/specificity, LR and SVM show intermediate performance, DT shows high specificity but poor sensitivity.

**Figure 4:**
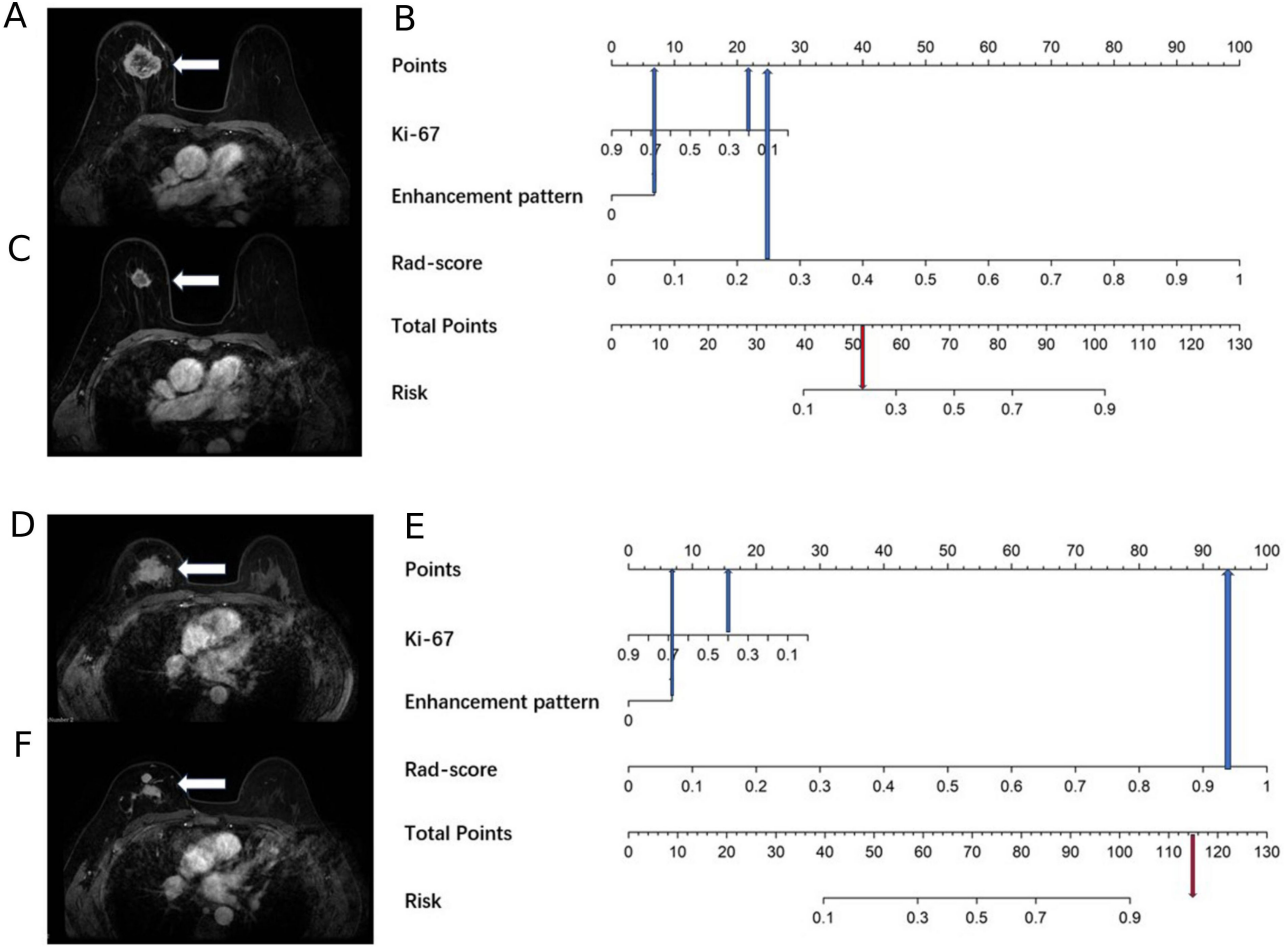
A 69-year-old female with right breast cancer and a Ki67 quantification of 20%, and a 40-year-old female with right breast cancer and a Ki67 quantification of 40%. **(A)** A 69-year-old female, the baseline axial contrast-enhanced MRI image, indicating a mass in the right breast (arrow) with heterogeneous enhancement. **(B)** A 69-year-old female, the nomogram, which predicts a risk value less than the threshold (0.38), indicating Type I shrinkage. **(C)** A 69-year-old female, the post-NAC MRI image, confirming Type I shrinkage. **(D)** A 40-year-old female, the baseline axial contrast-enhanced MRI image, indicating a mass in the right breast (arrow) with heterogeneous enhancement. **(E)** A 40-year-old female, the nomogram, which predicts a risk value greater than the threshold (0.38), indicating Type II shrinkage. **(F)** A 40-year-old female, the post-NAC MRI image, displaying centripetal shrinkage with satellite lesions, confirming Type II shrinkage.

**Figure 5:**
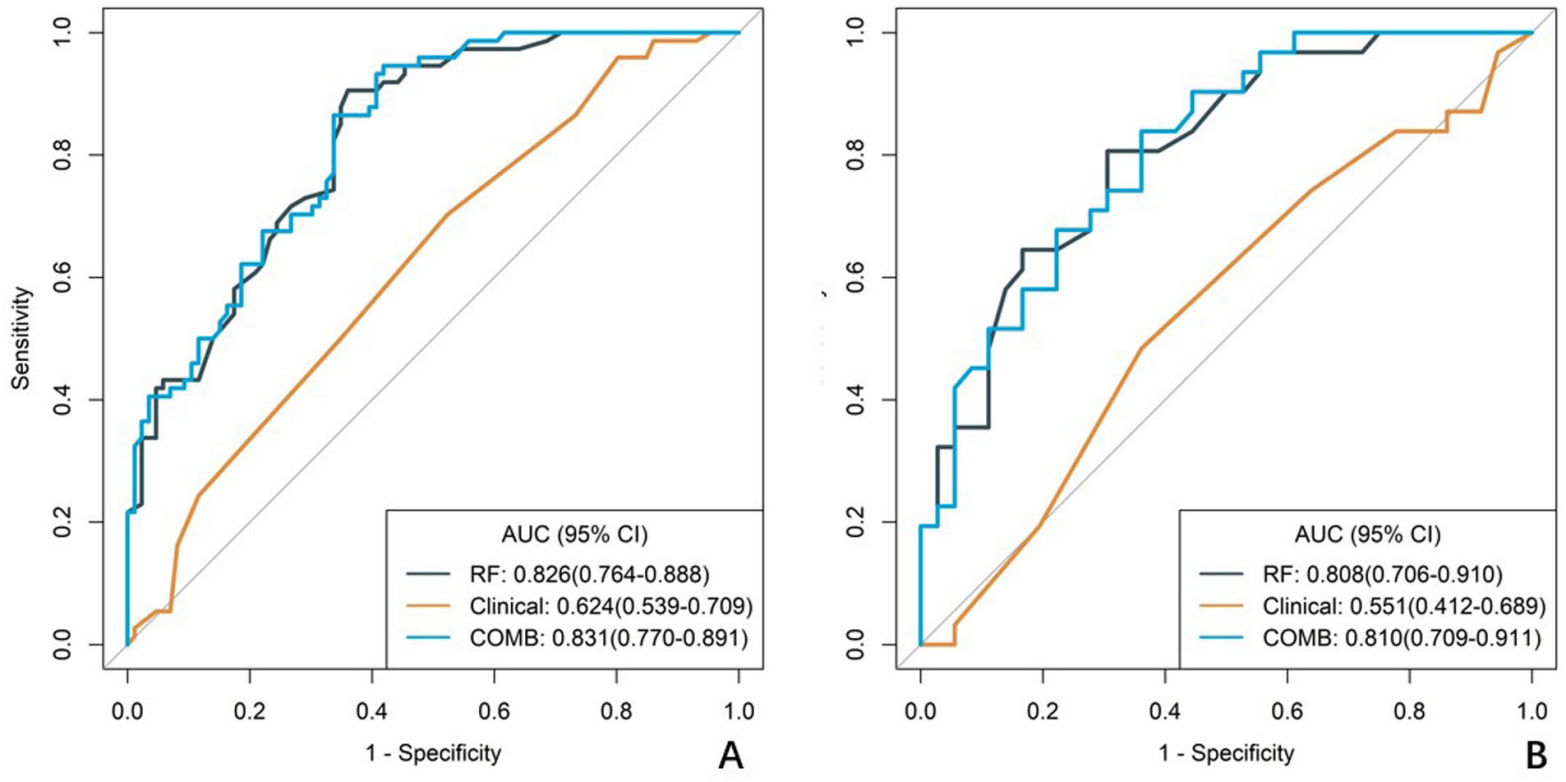
ROC curves of the clinical model, radiomics model, and combined model. **(A)** The ROC curves for the training cohort. **(B)** The ROC curves for the validation cohort. The diagnostic efficacy of the RF model in the training group and the validation group was slightly higher than the clinical model, but both were much higher than the combined model.

A total of 1688 radiomics features were extracted. After feature selection, 5 key features remained (**Figure 2**). Heatmap evaluation of the correlations among these features showed no strong correlations, as indicated by the Spearman correlation coefficient, suggesting no multicollinearity among the features (**Supplementary Figure S2**). Predictive models were constructed using four machine learning algorithms—Logistic Regression (LR), Support Vector Machine (SVM), Decision Tree (DT), and Random Forest (RF)—based on these 5 radiomics features. The predictive performance of each model is summarized in **Table 3** and **Figure 3**. The RF model exhibited the best predictive performance, with an AUC of 0.826 (95% CI: 0.764-0.888) in the training cohort and 0.808 (95% CI: 0.706-0.910) in the validation cohort. Therefore, the RF algorithm was ultimately used to construct the radiomics model for predicting tumor shrinkage patterns after NAC in HR+/HER2- breast cancer. A combined model was developed based on Ki67 quantification, enhancement pattern, and Rad-score, (**Supplementary Figure S3**) and a visual nomogram was created for individualized prediction. The diagnostic threshold was set at 0.38according to the maximum value of Youden’s index. A predicted value of<0.38 was classified as Type I shrinkage, while a value ≥0.38 was classified as Type II shrinkage (**Figure 4**). The AUC of the combined model in the training cohort was 0.831 (95% CI: 0.770-0.891) and 0.810 (95% CI: 0.709-0.911) in the validation cohort (**Table 4**, **Figure 5**).

**Table 3:** Predictive performance of radiomics models constructed using four machine learning algorithms.

Model	Training cohort				Validation cohort			
	AUC (95%CI)	Accuracy (95%CI)	Sensitivity (95%CI)	Specificity (95%CI)	AUC (95%CI)	Accuracy (95%CI)	Sensitivity (95%CI)	Specificity (95%CI)
LR	0.643 (0.558-0.729)	0.619 (0.539-0.694)	0.662 (0.500-0.770)	0.581 (0.372-0.698)	0.689 (0.562-0.817)	0.657 (0.531-0.768)	0.645 (0.290-0.839)	0.667 (0.361-0.778)
SVM	0.654 (0.569-0.739)	0.625 (0.545-0.700)	0.716 (0.514-0.825)	0.547 (0.372-0.640)	0.746 (0.628-0.865)	0.657 (0.531-0.768)	0.710 (0.387-0.968)	0.611 (0.444-0.806)
DT	0.676 (0.607-0.746)	0.650 (0.571-0.724)	0.419 (0.259-0.530)	0.849 (0.729-0.915)	0.632 (0.515-0.748)	0.642 (0.515-0.755)	0.419 (0.213-0.580)	0.833 (0.601-0.952)
RF	0.826 (0.764-0.888)	0.762 (0.689-0.826)	0.905 (0.669-0.959)	0.640 (0.430-0.733)	0.808 (0.706-0.910)	0.746 (0.625-0.845)	0.806 (0.516-0.935)	0.694 (0.389-0.861)

**Table 4:** Predictive efficacy of clinical models, imaging histology models, and combined models.

Model	Training cohort					Validation cohort		
	AUC(95%CI)	Accuracy(95%CI)	Sensitivity(95%CI)	Specificity(95%CI)	Accuracy(95%CI)	Sensitivity(95%CI)	Specificity(95%CI)	Accuracy(95%CI)
**Clinical**	0.624 (0.539-0.709)	0.581 (0.501-0.659)	0.703 (0.560-0.830)	0.477 (0.331-0.605)	0.551 (0.412-0.689)	0.537 (0.411-0.660)	0.742 (0.500-0.903)	0.361 (0.111-0.580)
**Radiomics**	0.826 (0.764-0.888)	0.762 (0.689-0.826)	0.905 (0.669-0.959)	0.640 (0.430-0.733)	0.808 (0.706-0.910)	0.746 (0.625-0.845)	0.806 (0.516-0.935)	0.694 (0.389-0.861)
**Combined**	0.831 (0.770-0.891)	0.756 (0.682-0.821)	0.865 (0.622-0.946)	0.663 (0.535-0.744)	0.810 (0.709-0.911)	0.701 (0.577-0.807)	0.774 (0.581-0.968)	0.639 (0.479-0.861)

### Comparison of different model performance

**Figure 6:**
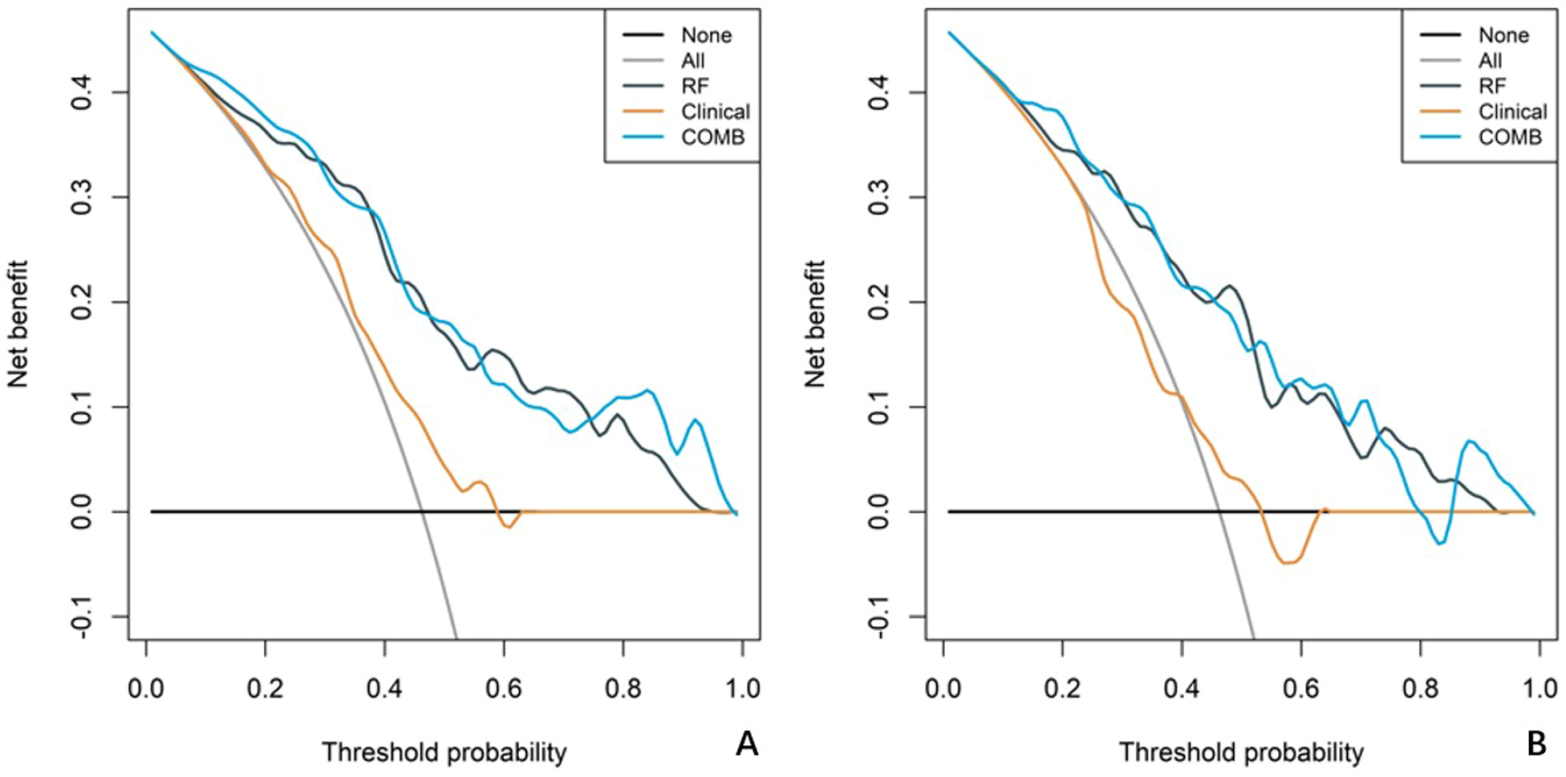
Decision curve analysis (DCA) curves for the clinical model, radiomics model, and combined model. The net benefit is plotted against the threshold probability for each model, comparing the performance of the different models. **(A)** The DCA curves for the training cohort. **(B)** The DCA curves for the validation cohort.

In both the training and validation cohort, the AUCs of the radiomics model and the combined model were significantly higher than that of the clinical model (all *p*<0.05). This method, also known as a sensitivity analysis, determines the contribution of each feature by measuring the decrease in model performance (e.g., AUC) when the values of that single feature are randomly permuted. A feature is considered more important if permuting its values leads to a larger drop in performance (**Supplementary Figure S4**). Although the AUC of the combined model was slightly higher than that of the radiomics model, the difference was not statistically significant (all *p*>0.05) (**Table 5**). The combined model demonstrated the best calibration performance in both the training set (*p*=0.011) and the validation set (*p*=0.13) (**Supplementary Figure S5**). Additionally, the clinical net benefit of the radiomics model and the combined model were higher than that of the clinical model (**Figure 6**).

**Table 5:** Comparison of clinical, imaging histology, and combined model AUCs.

Cohort	AUC (95%CI)			*P*	*P*	*P*
	Clinical (1)	Radiomics (2)	Combined (3)	(1vs2)	(1vs3)	(2vs3)
Training	0.624 (0.539-0.709)	0.826 (0.764-0.888)	0.831 (0.770-0.891)	<0.001	<0.001	0.617
Validation	0.551 (0.412-0.689)	0.808 (0.706-0.910)	0.810 (0.709-0.911)	0.004	0.001	0.905

## Discussion

HR+/HER2- breast cancer exhibits lower rates of pathologic complete response (pCR) and objective response after NAC compared to other molecular subtypes of breast cancer. To ensure patients benefit from NAC aimed at breast conservation, this study developed and validated a model based on clinicopathological characteristics, traditional MRI features, and radiomics features to predict tumor shrinkage patterns after NAC in HR+/HER2- breast cancer. The predictive performance of this model surpassed that of the clinical model, suggesting its potential utility in early identification of patients with poor shrinkage patterns and in timely adjustment of treatment strategies.

Univariable and multivariable regression analyses of clinicopathological characteristics and traditional MRI features revealed that Ki67 quantification was the only independent predictor of tumor shrinkage patterns after NAC in HR+/HER2- breast cancer. This finding is consistent with previous studies (11, 16, 17). Ki67 is a key marker for evaluating cellular proliferation activity, and its levels tend to rise with increasing tumor malignancy. It plays an important role in determining treatment plans and assessing prognostic risk in breast cancer. Theoretically, higher tumor proliferation activity correlates with greater sensitivity to cytotoxic drugs. Many studies suggest that patients with high Ki67 expression achieve better NAC outcomes (18–20). In this study, the Ki67 quantification was higher in the Type I shrinkage group compared to the Type II shrinkage group (30% vs. 20%).

Additionally, the proportion of tumors with homogeneous enhancement was higher in the Type I shrinkage group than in the Type II shrinkage group (13.953% vs. 4.054%). However, multivariate stepwise regression analysis indicated that homogeneous enhancement was not an independent predictor of tumor shrinkage patterns, possibly due to the limited sample size in this study. Uneven distribution of tumor cells and stroma, intra-tumoral hemorrhage, necrosis, and cystic changes are the main causes of heterogeneous enhancement, indicating significant tumor heterogeneity. This heterogeneity suggests differential responses to treatment within various tumor regions, leading to less ideal centripetal shrinkage patterns (21). Conversely, tumors with homogeneous enhancement exhibit lower heterogeneity, indicating similar treatment responses across tumor regions, thus more likely resulting in centripetal shrinkage patterns (22). Incorporating enhancement patterns into the clinical model improved its predictive performance.

Our results indicate that there are few clinicopathological and traditional MRI features with predictive value, and the predictive performance of the clinical model is suboptimal, with AUCs of 0.624 and 0.551 in the training and validation cohort, respectively. This suggests that current clinical routine examination techniques have limited value in predicting tumor shrinkage patterns after NAC in HR+/HER2- breast cancer. Radiomics, by deeply mining information contained in traditional imaging, can provide high-throughput, highly reproducible features that more accurately and objectively reflect tumor heterogeneity compared to traditional imaging (23). In this study, the radiomics model achieved AUCs of 0.826 and 0.808 in the training and validation cohort, respectively, while the combined model achieved AUCs of 0.831 and 0.810 in the training and validation cohort, respectively. The predictive performance of both the radiomics model and the combined model surpassed that of the clinical model. Although the AUC of the combined model was slightly higher than that of the radiomics model, the difference was not statistically significant, indicating that clinicopathological characteristics and traditional imaging features contributed little to enhancing the model’s predictive performance. Multivariable analysis also showed that the contributions of Ki67 quantification and enhancement pattern in the combined model were low (both *p*>0.05).

Although first-order features provide information on the gray-level distribution of the volume of interest, they do not describe information related to the relative positions of the various gray levels of the volume of interest. The five retained radiomics features in our study included one first-order feature and four texture features. Texture features mainly reflect the spatial distribution patterns of voxels and their correlation in the plane or in a certain direction, providing a quantitative representation of tumor heterogeneity, which significantly contributed to the predictive model (24).

There are some limitations to our study. Firstly, as a single-center, small-sample, retrospective study, there are inherent limitations. To mitigate selection bias, we applied strict, predefined inclusion and exclusion criteria to a consecutive cohort of patients. Furthermore, we ensured data integrity by only including patients with complete clinical, imaging, and pathological records, thus avoiding the need for data imputation. However, we acknowledge that this design has limited control over confounding variables. Therefore, the results need to be validated by multi-center, large-sample, prospective studies to confirm the robustness and generalizability of our model. Secondly, while the tumor shrinkage patterns were determined by two experienced radiologists through independent review followed by a consensus discussion to ensure accuracy, a formal quantitative metric for inter-rater reliability, such as the Kappa statistic, was not calculated. Future prospective studies should include such an analysis to formally validate the reproducibility of the classification criteria. Thirdly, the study aimed to predict tumor shrinkage patterns before NAC and considered the convenience of model use. Therefore, we only analyzed radiomics features from the initial phase of baseline MRI enhancement. Extracting features from multiple phases of enhancement or multiple sequences, such as DWI, might improve the model’s predictive performance.

## Conclusion

In conclusion, our study confirms that radiomics features based on baseline MRI can help early and accurately predict tumor shrinkage patterns after NAC in HR+/HER2- breast cancer patients, who generally have a lower response rate to NAC. This can assist in predicting the feasibility of breast conservation and timely adjustment of treatment strategies.

## Data Availability

The original contributions presented in the study are included in the article/**Supplementary Material**. Further inquiries can be directed to the corresponding authors.

## Supplementary material

Supplementary Figure 1 5-fold cross-validation procedure boxplot. Distribution of performance metrics (AUC, Sensitivity, Specificity) for each model across the 5 folds of cross-validation.

Supplementary Figure 2 Heatmap of the correlations between radiomics features. The Spearman correlation coefficients among the five selected radiomics features.

Supplementary Figure 3 Whether it is the training set or the validation set, RAD-SCORE can effectively distinguish the regression patterns of tumors after neoadjuvant chemotherapy. a: score-box-RF-train; b: score-bar-RF-train; c: score-box-RF-test; d: score-bar-RF-test.

Supplementary Figure 4 Feature Importance Analysis. Bar plot showing the results of the permutation feature importance analysis for the five radiomic features used in the final Random Forest model. The “Overall” importance score on the x-axis represents the model’s mean sensitivity to the permutation of each feature.

Supplementary Figure 5 Calibration curves of the clinical model, radiomics model, and combined model. The observed event percentages are plotted against the predicted event percentages for each model. The Hosmer-Lemeshow (HL) test values for each model are also indicated, demonstrating the calibration performance of the models. **(A)** The calibration curves for the training cohort. **(B)** The calibration curves for the validation cohort.
