# Supplementary figures and images for: Using baseline MRI radiomics to predict the tumor shrinkage patterns in HR-Positive, HER2-Negative Breast Cancer
Source: Front Oncol. 2025 Jul 30;15:1539644. doi: 10.3389/fonc.2025.1539644 (PMC12343230; doi:10.3389/fonc.2025.1539644)

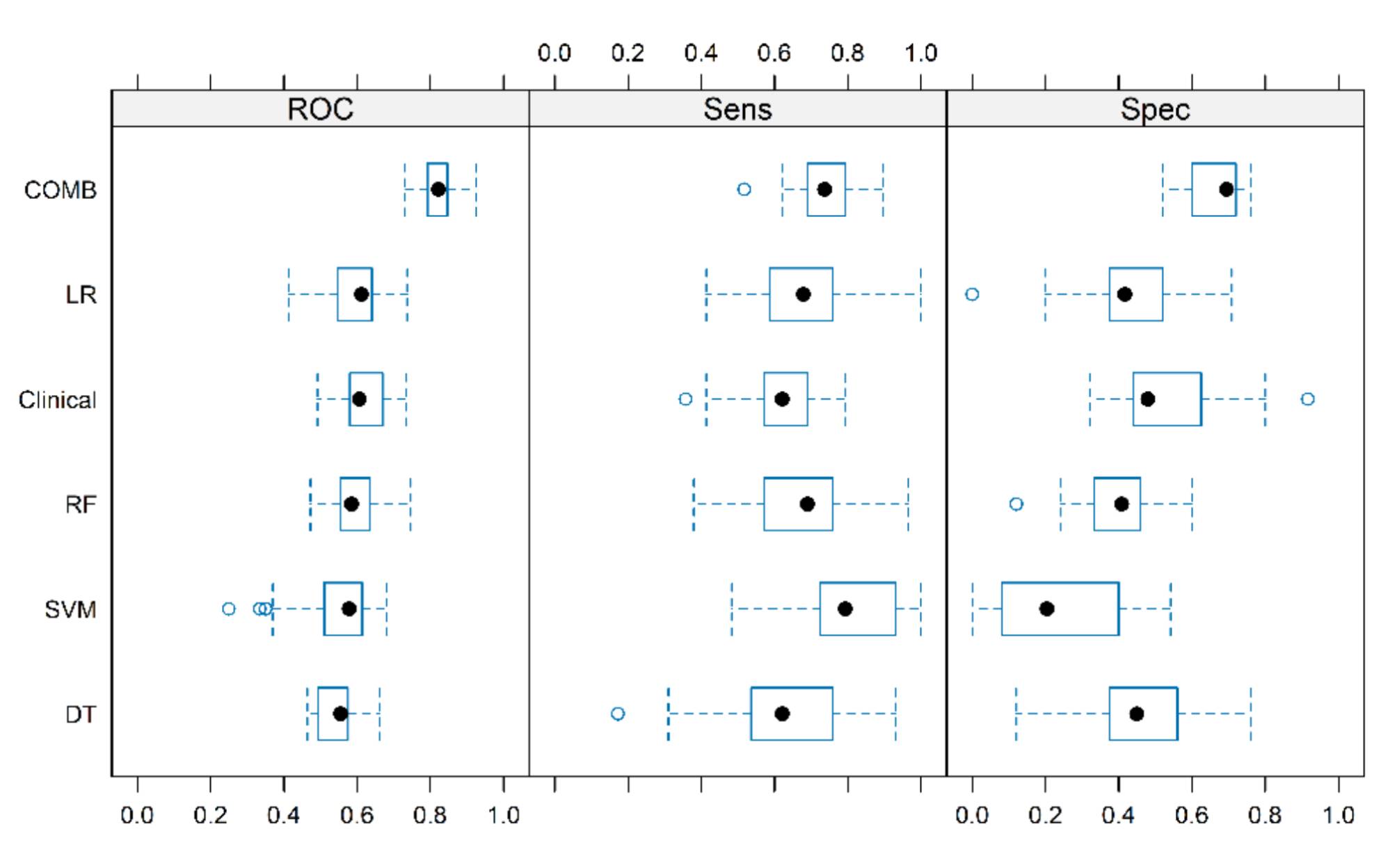

Supplement: Supplementary Figure 1 — 5-fold cross-validation procedure boxplot. Distribution of performance metrics (AUC, Sensitivity, Specificity) for each model across the 5 folds of cross-validation. [file Image1.jpg]

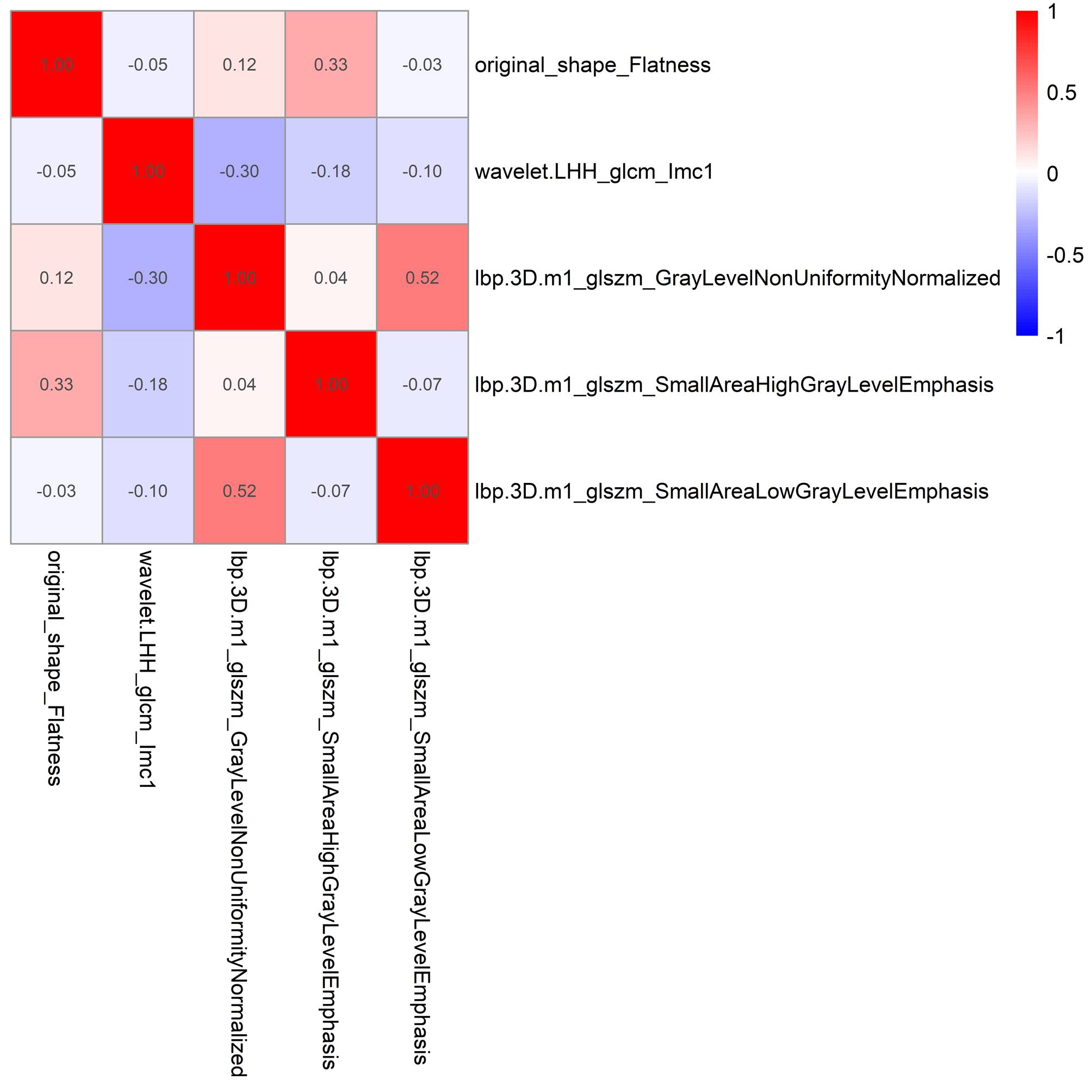

Supplement: Supplementary Figure 2 — Heatmap of the correlations between radiomics features. The Spearman correlation coefficients among the five selected radiomics features. [file Image2.jpg]

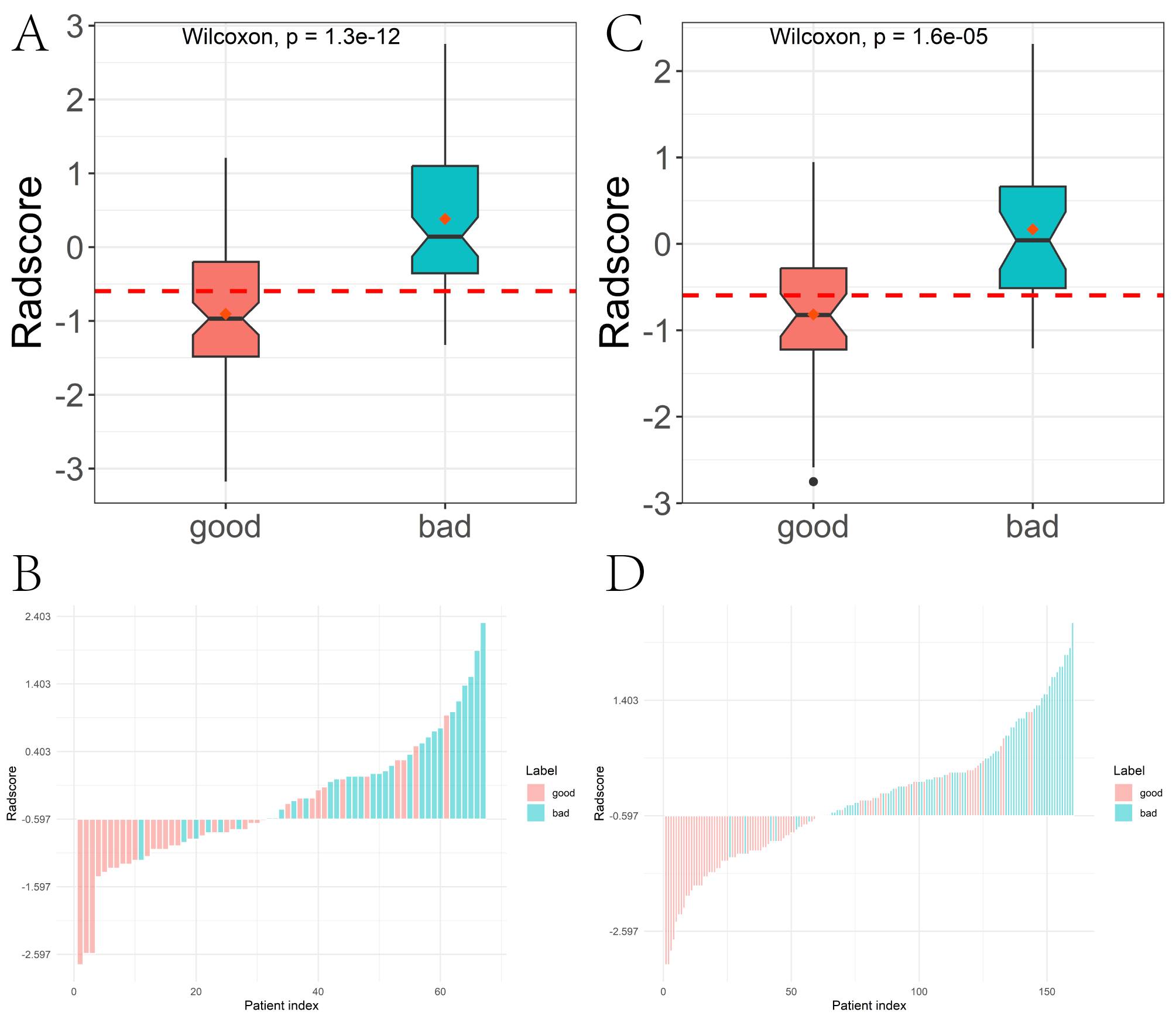

Supplement: Supplementary Figure 3 — Whether it is the training set or the validation set, RAD-SCORE can effectively distinguish the regression patterns of tumors after neoadjuvant chemotherapy. a: score-box-RF-train; b: score-bar-RF-train; c: score-box-RF-test; d: score-bar-RF-test. [file Image3.jpg]

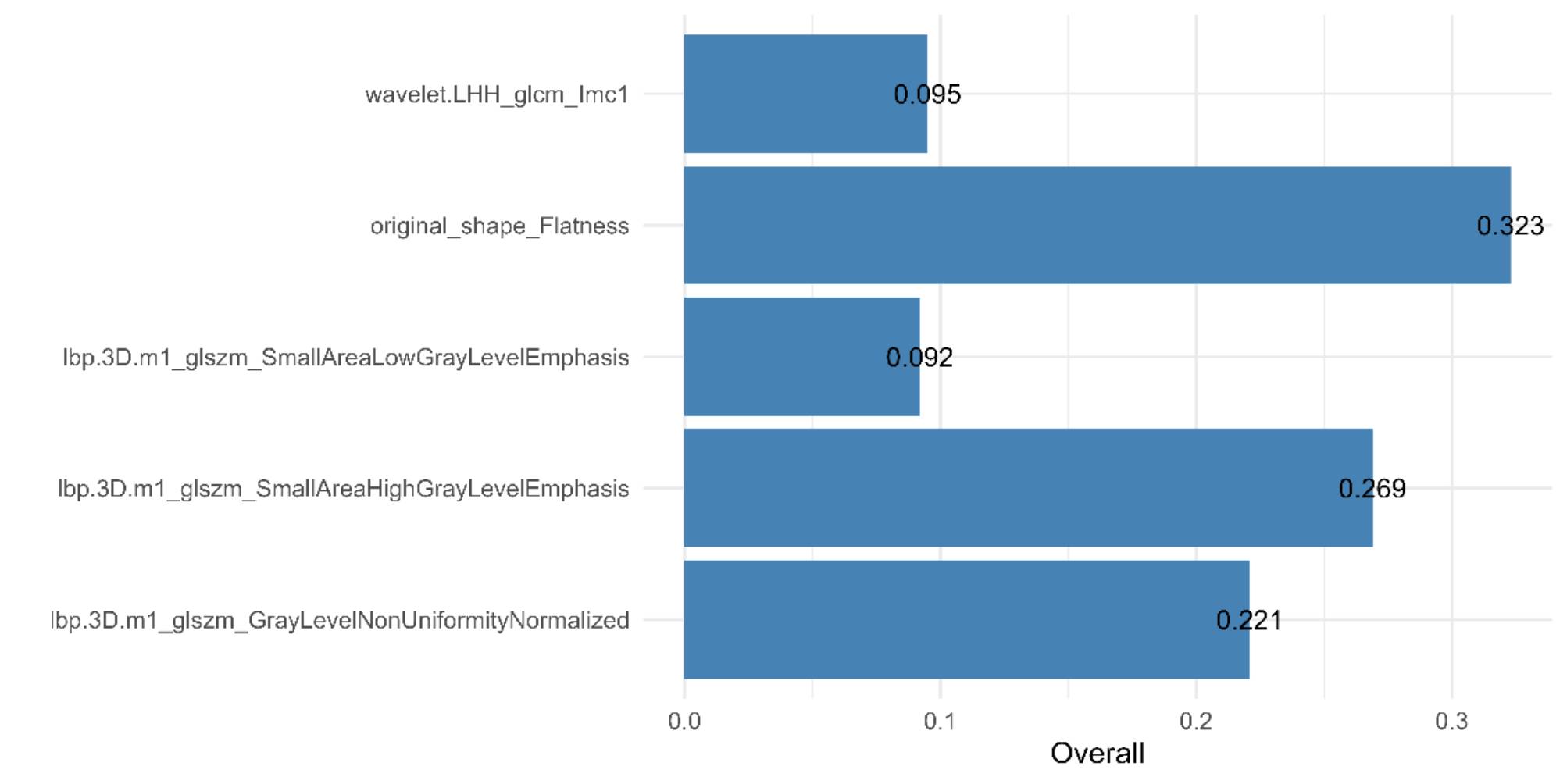

Supplement: Supplementary Figure 4 — Feature Importance Analysis. Bar plot showing the results of the permutation feature importance analysis for the five radiomic features used in the final Random Forest model. The “Overall” importance score on the x-axis represents the model’s mean sensitivity to the permutation of each feature. [file Image4.jpg]

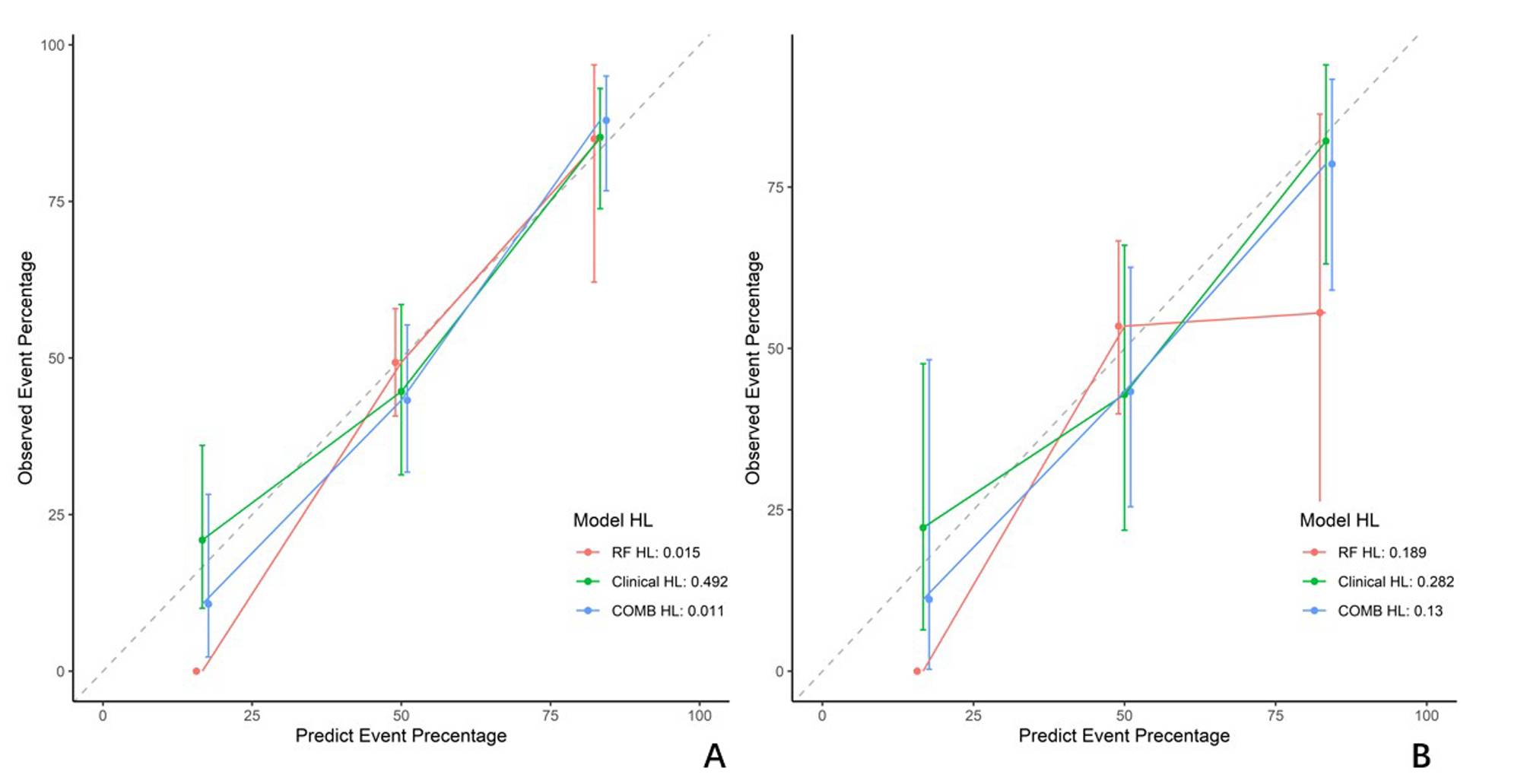

Supplement: Supplementary Figure 5 — Calibration curves of the clinical model, radiomics model, and combined model. The observed event percentages are plotted against the predicted event percentages for each model. The Hosmer-Lemeshow (HL) test values for each model are also indicated, demonstrating the calibration performance of the models. (A) The calibration curves for the training cohort. (B) The calibration curves for the validation cohort. [file Image5.jpg]
